# Construction methods and latest applications of kidney cancer organoids

**DOI:** 10.3389/or.2024.1434981

**Published:** 2024-11-12

**Authors:** Zhiqiang Li, Yanqiu You, Bingzheng Feng, Jibing Chen, Hongjun Gao, Fujun Li

**Affiliations:** ^1^ Medical College of Guangxi University, Nan Ning, Guang Xi, China; ^2^ Ruikang Hospital Affiliated to Guangxi University of Chinese Medicine, Nan Ning, China

**Keywords:** organoid, renal cell carcinoma, tumor microenvironment, co-culture, microfluidic device, drug screening, personalized treatment

## Abstract

Renal cell carcinoma (RCC) is one of the deadliest malignant tumors. Despite significant advances in RCC treatment over the past decade, complete remission is rarely achieved. Consequently, there is an urgent need to explore and develop new therapies to improve the survival rates and quality of life for patients. In recent years, the development of tumor organoid technology has attracted widespread attention as it can more accurately simulate the spatial structure and physiological characteristics of tumors within the human body. In this review, we summarize the main methods currently used to construct kidney cancer organoids, as well as their various biological and clinical applications. Furthermore, combining organoids with other technologies, such as co-culture techniques and microfluidic technologies, can further develop organoids and address their limitations, creating more practical models. This approach summarizes the interactions between different tissues or organs during tumor progression. Finally, we also provide an outlook on the construction and application of kidney cancer organoids. These rapidly evolving kidney cancer organoids may soon become a focal point in the development of *in vitro* clinical models and therapeutic research for kidney cancer.

## 1 Introduction

Renal cell carcinoma (RCC) is a major malignant tumor that originates from the renal tubular epithelial cells, ranking among the top ten malignant tumors worldwide (1). Renal cell carcinoma (RCC) represents a group of histologically and molecularly heterogeneous cancers which are originated from renal epithelium (2). RCC is further subdivided into subtypes including clear cell (accounting for 70%), papillary (accounting for 10%–15%), and chromophobe (accounting for 5%) (3). Globally, there are over 400,000 new diagnosed cases of RCC each year, resulting in approximately 175,000 deaths (4). Metastatic RCC has long been a chemotherapy-refractory malignancy (5). Prior to the development of targeted agents, renal cell carcinoma was one of the most drug resistant malignancies. For a long time, metastatic RCC has been difficult to treat effectively with chemotherapy, making it one of the most challenging malignancies to treat. In recent years, immune checkpoint inhibitors have become the standard first-line treatment for metastatic RCC (6), while anti-vascular endothelial growth factor (VEGF) drugs and mammalian target of rapamycin (mTOR) inhibitors are the standard treatment options (7). PD-1/PD-L1 inhibitors have been approved for the treatment of various advanced cancers, including RCC (8). Additionally, mTOR inhibitors like Everolimus and Temsirolimus have been approved for the treatment of resistant RCC (9). However, current treatments for RCC, including anti-angiogenic drugs, mTOR inhibitors, and immunotherapy combinations, can only extend the lives of many patients but rarely achieve complete remission, as almost all tumors progress within 2 years (10, 11).

Organoids, the self-organized three-dimensional tissue constructs, primarily stem from various types of stem cells—including pluripotent stem cells, fetal stem cells, and adult stem cells (12, 13). This unique technology can replicate the core functions, structures, and biological complexities of organs. The self-organizing process of organoids mimics the cellular interactions and signaling pathways in the body, providing researchers with a more physiologically relevant experimental environment (14). In recent years, by finely controlling the types, proportions, and culture conditions of cells, researchers have been able to guide cells to grow in specific environments and signaling pathways, successfully constructing various types of organoid models including liver (15), intestine (16), brain (17), lung (18) and gastric (19, 20). This development has greatly enriched the physiological relevance of experimental models, thereby promoting in-depth research on organ development, disease mechanisms, and drug responses. Particularly, patient-derived organoids (PDO) tumor models have provided important tools for studying tumor growth and treatment responses. PDO models are organoids established from patient-resected tumor tissues, capable of reproducing the histological and genetic characteristics of primary tumors. Compared with conventional 2D cultures, organoid cultures enable patient specificity in the model while recapitulating *in vivo* tissue-like structures and functions *in vitro* (13). In comparison to patient-derived xenografts (PDX), PDO models have shorter cultivation periods, higher success rates, and are suitable for high-throughput drug screening (21, 22). Spheroids are often cultured in a monolayer and therefore are often unable to reproduce the multicellular nature of tumors *in vivo*. Tumor spheroids are cultured under similar conditions to 2D cell lines. Compared to tumor spheroids, the multicellular characteristics of organoids imply that they are more patient-relevant and can predict patient responses to drugs (Table 1). These organoids are widely used in the study of various diseases, especially monogenic hereditary diseases. In addition, tumor organoids have become important tools for revealing the mechanisms of cancer initiation and progression. So far, various tumor-like organoids have been developed, including those for intestinal cancer (23), colorectal cancer (24), brain tumor (25), breast cancer (26), lung cancer (27), prostate cancer (28), and glioblastoma (29). However, even with a three-dimensional structure, organoids often lack vascular, immune components and physical cues. In addition to the multicellular structures known as organoids, the culture systems of organoids also include various growth factors selected based on their roles in kidney development, and the extracellular matrix (ECM) is also necessary for cell differentiation and orientation (30). Another issue with these organoids is the absence of nerves, blood vessels, and immune cells in the system. Some studies suggest significant differences in the transcriptome profiles between VHL-mutant cells and their primary tumor samples (31). However, further analysis shows that the differentially expressed genes in the tumor component mainly involve immune functions, suggesting that these genes are lost in VHL mutant cultures due to the lack of immune cells. Therefore, future research will focus on how to reactivate the immune response (32).

**TABLE 1:** Comparison of advantages and disadvantages of common cancer models.

	Cell lines	Patient-derived xenograft	spheroids	Organoids
Advantages	Less interference factors, easy synchronization, easier control of experimental conditions and easy gene manipulation	Preserve the microenvironment of parental tumor growth, High tumor similarity. Preserve tumor heterogeneity	Cells are generally more resilient and adapted to grow *in vitro*. Easy to operate and mass production. The cost of cultivation is low	Simulate the complexity of tumor microenvironments. High plasticity. The cultivation time is short. There are no ethical issues
Disadvantages	Partial or comp-lete loss of the characteristics of primary cells. Mutations may occur during lo-ngterm passage	The *in vivo* microenvironment cannot be fully simulated. Model building takes a long time. The success rate of modelbuilding is low	Single cell type, lack of complex microenvironments. The complexity of the tumor cannot be accurately mimicked	Lack of innate immune cells. No endocrine and neural regulation. The technology is not yet mature

**FIGURE 1:**
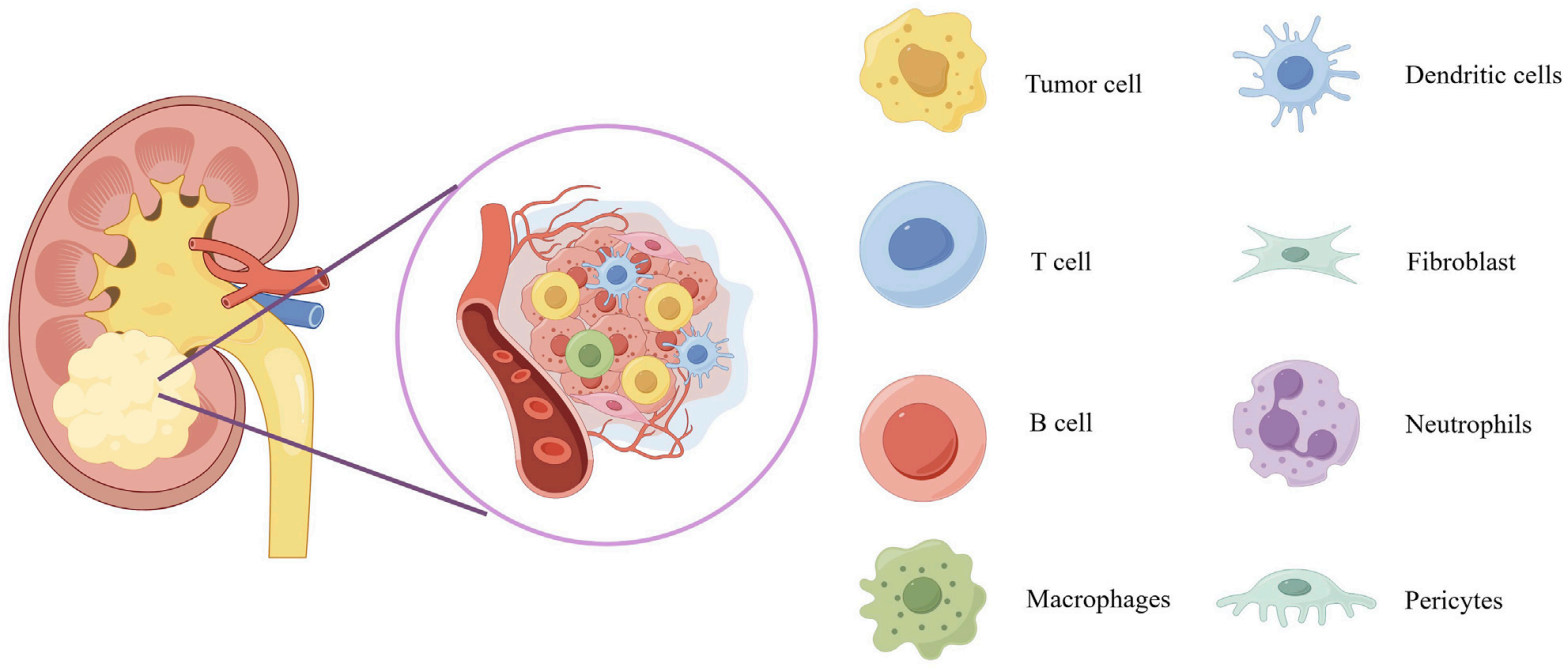
Schematic diagram of the tumor microenvironment of kidney cancer. The scheme provides an overview of the composition of tumor microenvironment. Overview of the tumor microenvironment composition displays blood vessels, tumor cells, dendritic cells, T cell, fibroblast, B cell, neutrophils, macrophages and pericytes.

The composition of tumors not only includes tumor cells themselves but also involves various non-tumor host components, collectively forming the so-called tumor microenvironment (TME). The TME plays a crucial role in promoting cancer initiation, tumor progression, and malignant cell metastasis. Its complexity stems from its diverse composition, including stromal source cells (such as fibroblasts and pericytes), vascular structures (including endothelial cells), and a diverse immune cell network (Figure 1). The latter encompasses both innate and adaptive immune cells, such as T cells, B cells, natural killer cells (NK cells), macrophages, dendritic cells (DCs), eosinophils, mast cells, and myeloid-derived suppressor cells (MDSCs), among others. Tumor organoid technology demonstrates tremendous potential for reconstructing three-dimensional structures and simulating heterogeneous cell components. However, there are limitations in replicating certain key aspects of the TME, such as tumor-specific biochemical/biophysical properties, anatomical scales, hierarchical blood/lymphatic vessel systems, and their fluid dynamics. These limitations can be overcome by integrating 3D bioprinting technology, creating models that are closer to clinical reality, comprehensive, and precise (33). On the other hand, microfluidics and bioprinting technologies show greater potential in manipulating complex and heterogeneous cellular aggregates. These technologies not only allow for fine adjustments to cell components and model geometry designs but also support high-throughput production and rapid processing, significantly enhancing the robustness, repeatability, and efficiency of tumor organoid production. These technological advances provide strong support for innovation and development in medical treatments (33).

In the foreseeable future, the primary trajectory of tumor organoid technology is implementation in clinical settings to enhance clinical pharmacotherapy and precision medicine. Notably, since 2016, tumor organoid technology has been incorporated into clinical trials, with 63 such trials officially registered with the FDA (34). Recently developed renal cancer organoids provide a highly representative *in vitro* model for the study of kidney cancer. These renal cancer organoids allow for the study of tumor development and the identification of cancer biomarkers within an environment that simulates the endogenous cellular and organ structures. Additionally, they facilitate the creation of biobanks for renal cancer organoids, drug screening, and personalized treatment.

This article introduces the composition of tumor organoids and various methods of generation (Table 2), elucidating the applications of tumor organoids in cancer research and therapy. Finally, we discuss the current challenges and future prospects for the broader application of tumor organoids.

**TABLE 2:** OrganoGel, growth factors, and culture media used in representative articles of kidney cancer organoids.

OrganoGel	Cell growth factors	Culture medium	Article
Matrigel	EGF, FGF, heparin, B27	DMEM/F12	(51)
Matrigel	HEPES, GlutaMAX, B27, A83-01, EGF, bFGF, Rock inhibitor	DMEM/F12	(52)
BME	B27, R-spondin, EGF, FGF-10, N-acetylcysteine, A83-01	NA	(57)
Matrigel	Recombinant human insulin, human transferrin, sodium selenite, Hydrocortisone, human recombinant Noggin, leucine, Y-27632	YRC	(58)
Matrigel	B27, Lue15 Gastrin, N-acetylcysteine, Recombinant human IGF-1, Recombinant FGF-2, Wnt 3a, R-spondin, Noggin, A83-01	DMEM/F12	(59)
Type I collagen	NA	Cancer media	(56)
Type I collagen	NA	RPMI	(60)
Matrigel	Primocin, EGF, CS-FBS, Y-27632, GlutaMAX	Hepatocyte culture medium	(61)
Matrigel	GlutaMAX, HEPES, B27, N-acetyl-L-cysteine, Nicotinamide, SB202190, Y-27632, Human EGF	DMEM/F12	(62)
Collagen gel	Wnt 3a, R-spondin1, HEPES, GlutaMAX, Niacinamide, N-acetyl-L-cysteine, B27, A83-01, SB-202190, EGF, Noggin, Normocin, IL-2	DMEM/F12	(63)

## 2 Cultivation method

**FIGURE 2:**
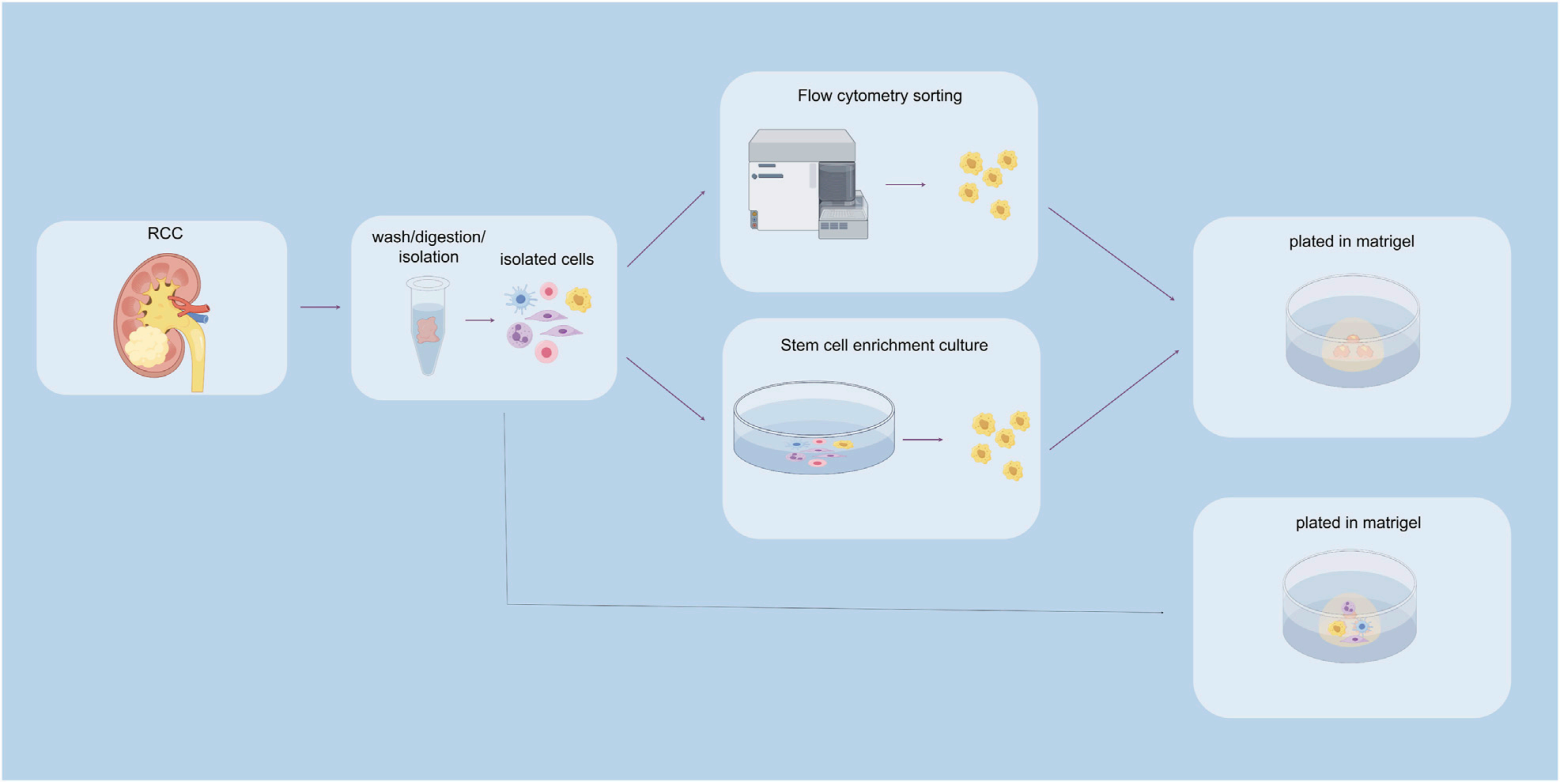
Construction of renal cancer organoids. The above pathways indicate stem cell sorting pathways, including flow cytometry sorting and stem cell enrichment culture sorting. The pathway below represents the unsorted stem cell pathway.

In the traditional sense, there are primarily two methods for culturing organoids: tissue chunk culture and isolated stem cell culture. Currently, most tumor organoid cultures utilize Matrigel as the culture matrix (Figure 2).

### 2.1 Three elements of tumor organoid construction

The construction steps of tumor organoids involve digesting fresh tumor tissue and culturing it within hydrogel domes extracted from basement membrane extracts (e.g., Matrigel, Cultrex BME), resembling the collagen-rich extracellular matrix found in human tissue. This complex structure allows cells to grow in a 3D manner, ensuring the simulation of cell-cell interactions within the body’s tissues, including all downstream effects of these interactions, such as cell signaling, metabolic alterations, and cell proliferation (35).

#### 2.1.1 Cell source

In the past decade, organoid-derived technologies have undergone significant development, particularly in the field of tumor research. These advancements include diverse approaches for selecting the source of tumor tissue and downstream processing methods. Tumor organoids can be derived from various tumor samples, such as primary tumors (36), metastatic lesions (23), circulating tumor cells (28) and tumor cells present in liquid effusions (37). The collection of these cells involves various techniques, including solid and liquid biopsies, surgical resection, and rapid autopsy.

The primary step in organoid preparation is the removal of non-epithelial tissues, such as fat or muscle, to a maximum extent. A portion of the tumor tissue is retained for subsequent molecular or biochemical analysis. The remaining tissue is then cut into small fragments ranging from 1 to 3 mm³ and subjected to enzyme digestion until clusters containing several to tens of cells form. These cell clusters or individual cells are subsequently filtered through a cell strainer with a pore size of 100 μm to remove any undigested tissue fragments, and they are seeded into 3D extracellular matrix (ECM) hydrogels such as Matrigel, Geltrex, or basement membrane extracts (BME).

Lastly, these cells are cultured in specific media supplemented with growth factors to promote their growth and development. Through this series of meticulous procedures, researchers are able to cultivate organoids that mimic the actual tumor environment, thereby opening new perspectives in cancer research and treatment.

#### 2.1.2 Matrigel

ECM is a complex three-dimensional network surrounding cells, primarily composed of fibrous proteins such as collagen, elastin, fibronectin, and laminin, as well as protein polysaccharides including chondroitin sulfate, heparan sulfate, keratan sulfate, and hyaluronic acid, forming the structural framework of most tissues (38). During tumor evolution, significant changes in the composition and content of ECM occur, influencing the behavior of tumor and stromal cells, such as proliferation and migration. Studies indicate that upregulation of ECM remodeling-related genes is closely associated with increased mortality rates in patients with breast cancer, lung cancer, and gastric cancer (39, 40). Collagen, as the most abundant component of ECM, not only provides structural integrity and strength but also plays a crucial role in regulating the tumor microenvironment (41).

Organoids, especially tumor models, are typically cultured in basement membrane extracts (BME) derived from Engelbreth-Holm-Swarm mouse sarcoma cells, such as Matrigel, Geltrex, or Cultrex BME. Although these natural ECM extracts are rich in proteins that promote organoid formation, their complex and variable composition, along with potential immunogenicity, pose challenges (42). Therefore, synthetic hydrogels have been proposed as a solution. They are 3D polymer networks capable of mimicking the composition and mechanical properties of natural ECM while allowing the permeation of oxygen, nutrients, metabolites, and waste. Polyethylene glycol (PEG) is one widely used material due to its excellent hydrophilicity and tunable stiffness. By combining synthetic polymers with natural ECM components, biohybrid hydrogels are produced, combining the advantages of both sides and providing an ideal supportive environment for cells.

Three main types of biohybrid hydrogels—photopolymerized hydrogels, enzyme-mediated polymerized hydrogels, and click chemistry cross-linked hydrogels—show excellent performance in culturing tumor organoids (43). For example, incorporating fibronectin or hyaluronic acid into methacrylated gelatin hydrogels significantly improves organoid growth. This suggests that constructing artificial ECMs suitable for different cancer types of tumor models is feasible, but overcoming numerous challenges is still necessary to find the ideal choice for each application.

#### 2.1.3 Cell growth factors

In the field of tumor organoid culture, Wnt proteins and their signaling pathways play a central role. The Wnt/β-catenin signaling pathway is activated in various types of tumors and is closely associated with tumor formation, proliferation, migration, and metastasis (44). The Wnt signal influences the cell cycle and guides the allocation of new cells, promoting the formation of organized body structures rather than amorphous structures (45). This process involves not only changes in gene expression but also affects cell cytoskeleton and mitotic spindles and is crucial for maintaining the self-renewal capacity and pluripotency of tumor stem cells (46). By regulating the activity of the Wnt signal, the state of tumor stem cells can be controlled, thereby influencing the development of tumor tissue.

R-spondin proteins, as enhancers of the Wnt signal, bind to the Lgr5 receptor, blocking the degradation of Wnt receptors and maintaining the continuous activation of the Wnt signal, which is essential for maintaining stem cells. Studies have shown that R-spondin-1 effectively promotes Wnt-dependent proliferation of intestinal crypts (47). Additionally, Wnt signaling alone is not sufficient to sustain the self-renewal of Lgr5+ stem cells; R-spondin-mediated stem cell amplification is required to confer self-renewal capability (48).

Noggin protein is an important antagonist of the BMP signal. By binding to members of the BMP family, it inhibits their interaction with surface receptors and blocks BMP signal transmission. This action changes the cell’s response to differentiation signals, leading to the differentiation of specific cell lineages. In intestinal organoid culture, the removal of Noggin leads to the loss of Lgr5 expression and cessation of proliferation in organoids (16).

EGF is a critical growth factor that promotes rapid proliferation and growth of cells in organoids by binding to EGFR. Activated EGF signaling pathways, such as MAPK, PI3K/AKT, and JAK/STAT, collectively regulate cell proliferation, differentiation, and survival. EGF also influences the expression of specific genes that are typically involved in cell cycle regulation, thereby promoting changes in cell function and the formation of tissue structures. For example, long-term cultivation of intestinal organoids relies on the activation of EGF signaling, while in certain organoids, disruption of the negative feedback mechanism of EGF signal transduction reduces the dependence on EGF, revealing the complex role of EGF in promoting organoid growth (49).

**FIGURE 3:**
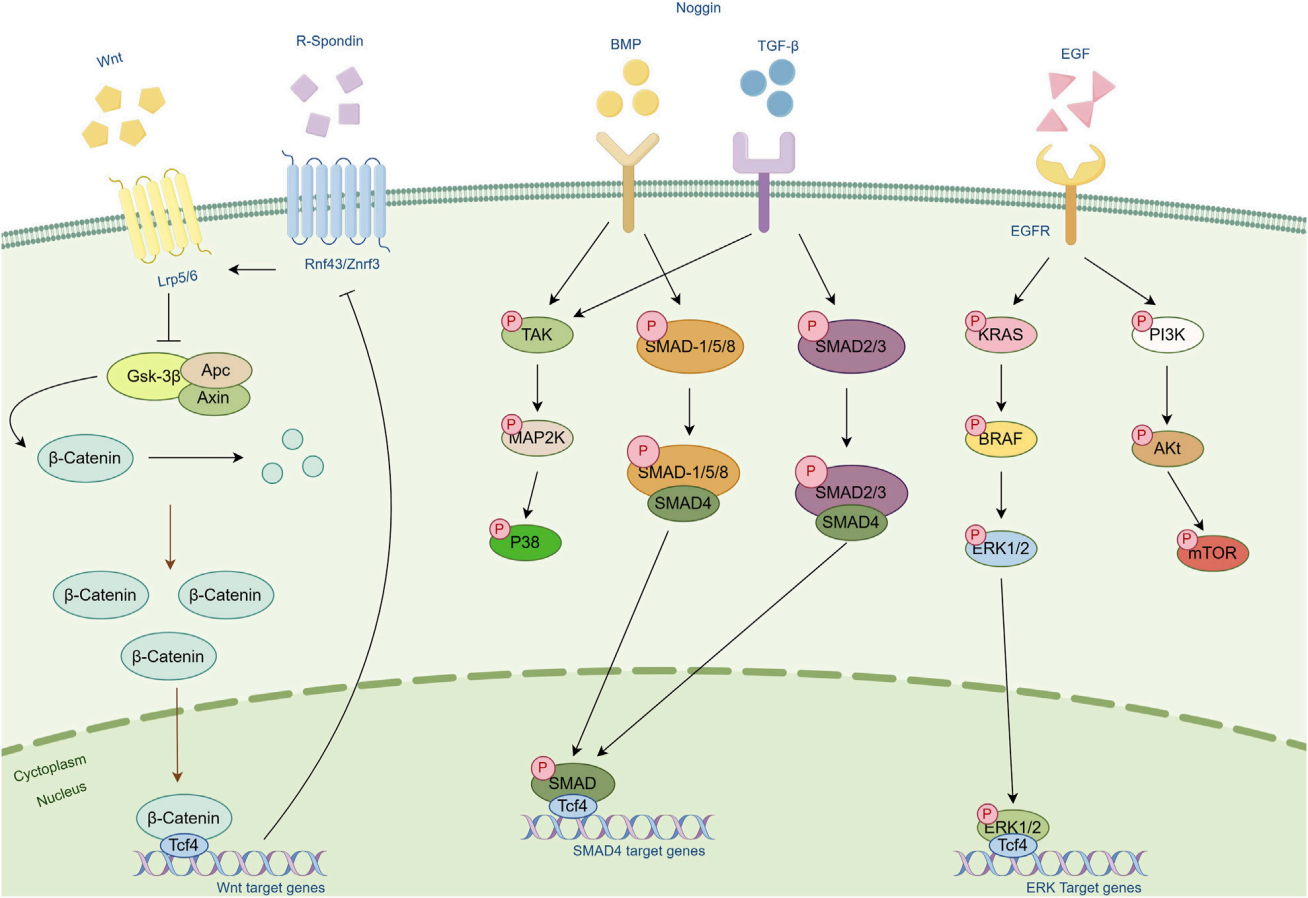
Schematic diagram of the cellular pathways of several important cytokines. Cytokines such as Wnt, R-Spondin, Noggin and EGF and their associated pathways are included in the figure. Arrows represent modifications of the protein or activation of another protein or mechanism. Bar-headed lines represent inhibitory activity.

In summary, Wnt proteins, along with R-spondin, Noggin, EGF, and other molecules, play crucial roles in tumor organoid culture. They regulate signal transduction pathways and cell behavior, collectively promoting the formation and development of tumor tissues (Figure 3).

### 2.2 Sort stem cell cultures

Cancer stem cells (CSCs), a type of stem-like cells found in certain tumor tissues, play a crucial role. These cells not only possess the ability for self-renewal and unlimited proliferation but also can migrate to specific tissues and resist the effects of toxic chemical factors. Of concern is that even with treatments like radiation therapy, chemotherapy, or immunotherapy, tumors may show significant improvement, but as long as CSCs persist, there remains a possibility of tumor recurrence. This underscores the decisive role of CSCs in the treatment resistance and metastatic potential of tumors, pointing directly to the fact that complete cure remains elusive unless treatment approaches can specifically target this subset (50, 51). Although the role of CSCs is pivotal, research into the characteristics of tumor stem cells, especially in diseases like ccRCC, has yet to be fully elucidated. This highlights one of the crucial barriers to breakthroughs in cancer treatment: the presence of CSCs makes tumors difficult to eradicate.

In practical research, by isolating CSCs from tumor tissues and constructing organoids, researchers can gain insights into the characteristics of these cells. For example, Fendler and colleagues successfully constructed organoid models of clear cell renal cell carcinoma by isolating CSCs from kidney cancer tissues. As these organoids grew, the gradual differentiation of CSCs was observed, ultimately resembling the content of tumor tissues *in vivo* (51). Furthermore, Grassi et al similarly constructed kidney cancer organoid models that recapitulate tumor characteristics using CSC culture methods (52). Regarding the methods for isolating tumor stem cells, two completely different approaches were employed. Fendler et al utilized flow cytometric sorting to isolate tumor stem cells, using markers CXCR4+, MET+, and CD44^+^. They found that the CXCR4+MET+CD44^+^ cells exhibited a higher capacity for self-renewal and were highly enriched in tumors with lymphatic infiltration and distant metastasis (51). In contrast, Grassi et al employed an enrichment culture method to isolate tumor stem cells. They cultured the cell suspension obtained from digestion and dissociation in serum-free stem cell enrichment medium for 72 h (52).

### 2.3 Unsorted stem cell cultures

The composition of tumor tissue is far from singular; it contains not only tumor cells but also various immune cells, tumor-associated fibroblasts, as well as elements such as blood vessels and nerve fibers. When cultivating tumors *in vitro*, replicating the tumor microenvironment is crucial in addition to the tumor cells themselves. Constructing organoids directly from tumor tissues obtained through surgery or biopsy allows for the inclusion of multiple cell types from the tumor microenvironment, thereby more accurately simulating the *in vivo* conditions.

However, challenges arise with this approach. In standard organoid culture media, non-malignant cells often proliferate faster than malignant cells, potentially leading to overgrowth of cells surrounding the tumor and even, in some cases, inhibiting the growth of malignant tumor cells. A study involving 59 patients with lung cancer organoids found that contamination with healthy airway cells resulted in excessive growth in 58% of the organoids (53). This highlights the challenge faced in directly culturing most tumor tissue blocks, where conventional culture media lack necessary supplements such as noggin, R-spondin, and Wnt 3a. However, studies on kidney cancer organoids suggest that enriched culture media containing these supplements can support the growth of normal renal epithelial organoids (54).

Further research, such as the work by Joon et al., successfully established organoids of ccRCC using tissue block culture methods from surgically resected kidney cancer tissues. They found a significant increase in the expression of tumor markers, indicating a more precise replication of the characteristics of *in vivo* kidney cancer tissues. It’s noteworthy that organoids derived from ccRCC must be cultured without ROCK inhibitors, as these inhibitors can impair cell proliferation due to synthetic lethal effects associated with VHL defects (55).

However, research by Agata found that unsorted kidney cancer cells displayed larger sphere sizes in organoid culture, and exclusive overgrowth of non-malignant cells was not observed in specific culture media (56). This finding contradicts previous observations, suggesting that the cellular dynamics in tumor tissue culture are more complex than currently understood, requiring further research to reveal the underlying mechanisms.

## 3 Advanced cultivation method

### 3.1 Co-culture

Compared to cell line culture systems, tumor organoids indeed provide a more realistic model of tumor tissue. However, given the absence of immune cells, nervous system, or mature TME within tumor organoids, co-culture models offer a good solution. Co-culture models of tumor organoids can drive organoid formation through direct or indirect interactions between specific cell types within the tumor. Additionally, they can be used to study immune crosstalk between tumor organoids and specific cells (64). Next, I will specifically describe co-culturing specific cell types with cancer organoids.

#### 3.1.1 Co-culture with immune cells

With the continuous advancement of immune checkpoint inhibitor therapy in cancer treatment, this field has witnessed a significant shift in treatment paradigms. Nonetheless, the issue of resistance remains a major challenge for immunotherapy, with tumor heterogeneity and resistance to treatment further complicating the early development of novel immunotherapies. Specifically, the heterogeneity within tumors and between tumors, along with varying immune responses among patients, make the development of these therapies even more complex.

Faced with this challenge, researchers urgently need new approaches to address tumor heterogeneity among patients. Recent studies have indicated the potential feasibility of co-culturing PDOs from renal cancer with immune cells in immuno-oncology research. For instance, researchers like Dekkers et al. from the Netherlands have developed a co-culture model of cancer cells with engineered T cells, revealing a cluster of T cell behavior with robust and sustained cytotoxicity, referred to as “super phagocytes.” This analysis exposes the limitations of single-cell analysis techniques in exploring dynamic cell behavior and proposes a novel integrated platform aimed at a deeper understanding and optimization of tumor-targeted treatment mechanisms (65). Additionally, models of co-culturing tumor organoids with endogenous T cells developed by Cattaneo not only amplify tumor-reactive T cells but also assess the activity of these T cells against matched tumor cells (66). Further, Neal et al have developed an air-liquid interface co-culture model, significantly preserving various endogenous immune cell types such as macrophages, B cells, and NK cells, maintaining the complex structure of the original tissue rather than just adding clonally expanded or TCR-engineered tumor-infiltrating lymphocyte (TIL) populations (67). Through such models, Esser et al successfully established co-cultures of tumor organoids with immune cells from 42 cases of renal cancer tissues collected from patients, achieving a success rate of 72%. They examined the response of PDOs to cabozantinib and nivolumab treatments, finding that PDOs from different patients exhibited varying responses to the same drug, with most PDOs showing a good response to only one of the treatments, similar to the results observed in actual treatment processes (22). This demonstrates that PDO models can accurately replicate the tumor microenvironment within patients’ bodies, providing an important research tool for overcoming tumor heterogeneity and enhancing the effectiveness of immunotherapy.

#### 3.1.2 Co-culture with tumor-associated fibroblasts

Tumor-associated fibroblasts (CAFs) hold a significant position within the kidney cancer tissue, abundantly present and establishing a dense ECM that acts as a physical barrier significantly reducing the efficacy of drug delivery to tumor cells (68). An increasing body of research reveals the role of CAFs in facilitating tumor progression, spread, and treatment resistance. Specifically, CAFs play a crucial role in tumor progression by affecting the synthesis of the matrix and growth factors, regulating tumor metabolism, recruiting immune cells, and enhancing the chemoresistance of tumor cells. In RCC, CAFs not only promote tumor development but are also associated with resistance to treatments such as mTOR inhibitors and VEGF-TKIs (vascular endothelial growth factor receptor tyrosine kinase inhibitors) (69).

Despite the importance of interactions between the tumor and the extracellular matrix, research in this area has been relatively slow. Most existing tumor organoid models lack CAFs, limiting their applicability. However, recent co-culture models have provided potential solutions to this challenge. A notable example is researchers screening for CD44^+^ cells using flow cytometry and co-culturing these cells with CAFs in Matrigel. This co-culture method not only enhanced the organoid-forming capability of CD44^+^ cells but also found that this enhancement was blocked when the production or uptake of lactate in CAFs or CD44^+^ cells was inhibited. Additionally, lactate treatment promoted the organoid-forming capability of CD44^+^ cells and increased the protein levels of CD44 and OCT-4 in oral squamous cell carcinoma (OSCC) organoids (70).

Further studies showed that Naruse and colleagues successfully prepared colorectal cancer (CRC) organoids and their corresponding CAF-co-cultured CRC organoid models, and compared their expression profiles. The results revealed that 177 genes, including those in the REG and DUOX families, were significantly upregulated in the co-culture model, with many of these upregulated genes having oncogenic functions, highlighting the significant role of CAFs in tumor progression.

In summary, CAFs play an extremely important role in tumor growth, spread, and treatment resistance, and recent research using co-culture models further reveals the complexity and significance of the interactions between CAFs and tumor cells. These findings offer new perspectives and possibilities for developing novel therapeutic strategies targeting the tumor microenvironment.

### 3.2 Microfluidic device

In TME, aside from immune cells, tumor-associated fibroblasts, and vascular structures, various physicochemical conditions such as oxygen concentration, pH, and shear forces play crucial roles in tumor development and metastasis. For instance, higher fluid shear stress (FSS) is associated with tumor cells releasing pro-angiogenic and pro-inflammatory factors and can induce necrosis of circulating breast cancer cells (71, 72). Particularly in the kidney, FSS values vary significantly in different regions, which is of paramount importance for studying renal diseases (73). However, traditional culture and co-culture models struggle to simulate these complex physicochemical conditions.

Recently, organ-on-chip platforms based on microfluidic technology have emerged, offering a novel pathway for research by mimicking the structure and function of human organs. These chips consist of tiny fluidic channels and cell culture chambers capable of simulating physiological interactions and generating the necessary concentration gradients and controlling fluid shear stress through microfluidic systems, thereby achieving precise distribution of cell types and simulation of biophysical conditions. Additionally, this technology ensures continuous supply of oxygen and nutrients, enhancing the model’s ability to simulate tumor physiology (74). On the other hand, microfluidic devices are powerful tools for TME modeling, as multiple factors within the TME can be individually and accurately controlled on the microfluidic platform. They can be easily customized according to different experimental requirements, including multiple chambers and channels with varying structures, allowing precise control over cell distribution and fluid flow in the microfluidic platform. This includes 3D cell culture, extracellular matrix, co-culture of cells, vascular systems, chemokine gradients, hypoxia, and biophysical forces (75). In complex 3D *in vitro* models of kidney cancer, a functional vascular system is crucial. Vessels can be formed within the microfluidic device by seeding endothelial cells in the channels or by exploiting the self-organizing properties of endothelial cells co-cultured with fibroblasts. For example, researchers have used microfluidic chips to simulate the oxygen concentration gradients within the tumor microenvironment. By controlling the supply and consumption of oxygen within the microfluidic channels, they generate varying oxygen gradients, thus mimicking the distribution of oxygen within tumor tissues. This model can be used to study the growth and metastatic potential of tumor cells under different oxygen conditions. When applied to kidney-like organs, FSS can lead to more mature differentiated structures, offering higher sensitivity and better physiological function towards nephrotoxic compounds (76). Furthermore, Ozcelik et al designed a microfluidic device that co-cultures patient-derived kidney cancer cells with mesenchymal stem cells in alginate hydrogel. They used cisplatin as a chemotherapeutic agent and subsequently measured the gene expression levels of CXCR4 and CXCL12. The experimental data indicated that the kidney cancer organoids produced by this device could be used to measure the effect of drugs and the response of the tumor microenvironment (77).

In summary, organ-on-a-chip devices based on microfluidic technology offer a more precise and controllable method for simulating the tumor microenvironment. They aid researchers in better understanding the development and metastatic mechanisms of tumors and provide important references for developing new therapeutic strategies.

## 4 Application of renal cancer organoids

**FIGURE 4:**
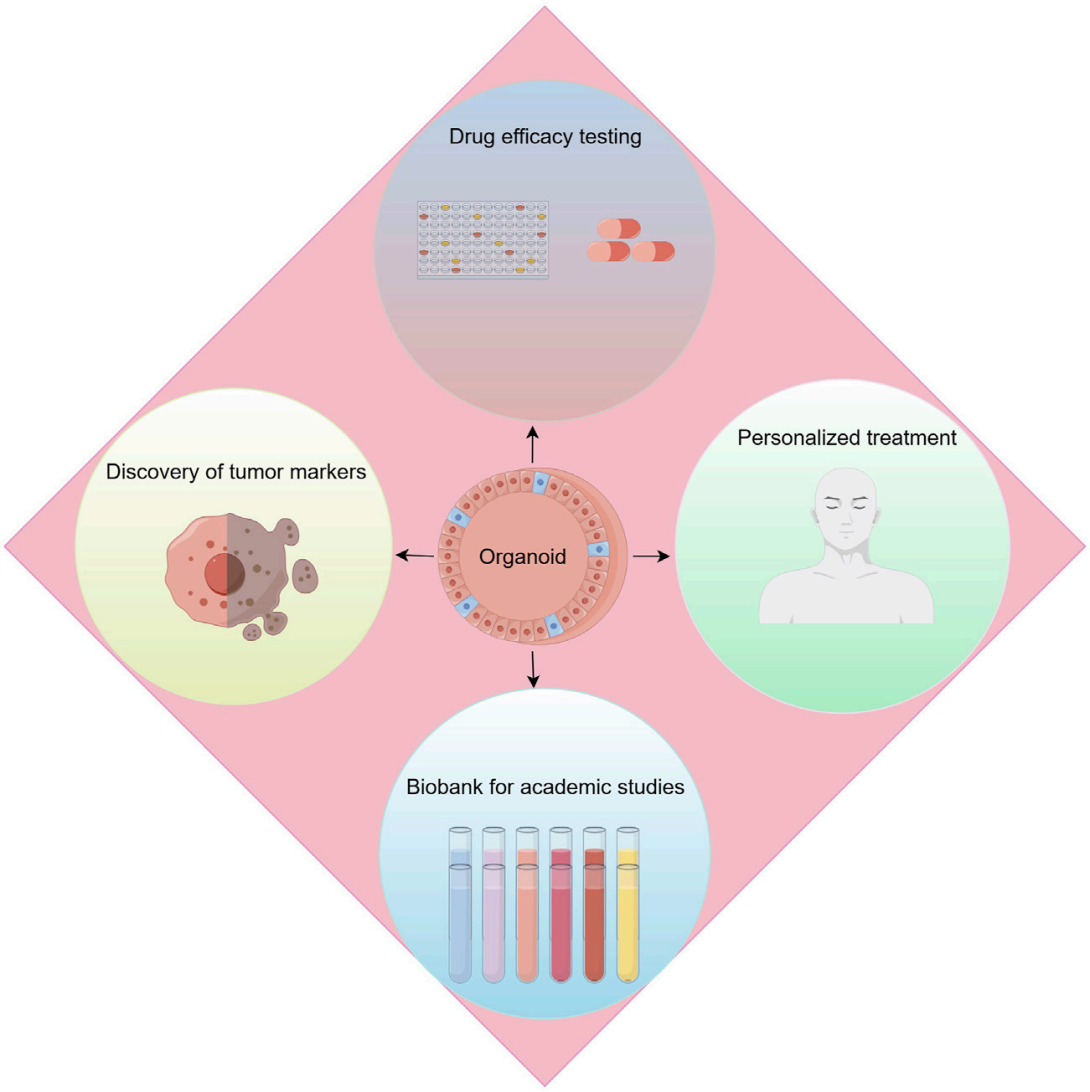
Applications of renal cancer organoids. Applications include drug efficacy testing, discovery of tumor makers, personalized treatment and biobank for academic studies.

With the rise of precision medicine, the demand for research methods in kidney cancer is constantly evolving, especially in precise simulation of the renal cancer microenvironment, exploration of tumor intrinsic heterogeneity, and assessment of drug effects. Against this backdrop, kidney cancer organoid technology has emerged, bringing revolutionary advances to the field of research (Figure 4).

Kidney cancer organoid technology, this advanced biotechnological approach, allows researchers to cultivate patients’ own tumor cells *ex vivo*, forming three-dimensional structures that simulate the real renal cancer microenvironment. The advantage of this method lies in its ability to maintain the tumor’s original biological characteristics and heterogeneity, while also reproducing the complex interactions between tumor cells and their microenvironment, thus providing a powerful tool for in-depth exploration of the development mechanisms of kidney cancer. More importantly, the use of kidney cancer organoids has greatly promoted the development of personalized treatment plans, enabling doctors to tailor the most appropriate treatment strategies based on the specific organoids’ response to drugs.

The continuous advancement of technology has made the construction and application of kidney cancer organoids more refined and efficient, becoming a valuable platform for kidney cancer research, drug development, and optimization of treatment strategies. This patient-centered research model not only shortens the drug development cycle and reduces costs but also has the potential to lead innovation in kidney cancer treatment, providing patients with more precise and effective treatment options.

### 4.1 Drug screening

The process of translating a new drug from concept to clinical application is both lengthy and costly, typically requiring 10–15 years and billions of dollars in research and development expenses. This pathway includes drug discovery, preclinical research, clinical trials, and regulatory approval, each step demanding significant amounts of time, funds, and resources. Particularly, drug development faces a remarkably high risk of failure, with a success rate of only about 5% from initial screening to approval.

Against this backdrop, kidney cancer organoids, as an emerging technology, demonstrate unique advantages in the process of new drug screening. Compared to traditional two-dimensional cell cultures and animal models, kidney cancer organoids can more accurately simulate the microenvironment within the human body, thus providing more precise assessments of drug effects. Utilizing kidney cancer organoids to early screen out drugs with poor efficacy or toxic side effects can effectively reduce the risk of failure in later-stage clinical trials.

Specifically, researchers have successfully established representative kidney cancer organoids from patient-derived sources, particularly those with metastatic renal cell carcinoma (tRCC) harboring the PRCC-TFE3 fusion gene (59). These organoids not only capture the complexity and heterogeneity of tumors but also can be used for high-throughput drug screening experiments. Through the use of high-throughput screening techniques to test 1816 compounds, the research team has identified several potential drugs, such as axitinib, crizotinib, and JQ-1, which can effectively inhibit the cell cycle of kidney cancer organoids and induce apoptosis. Furthermore, Grassi et al (52) investigated the effects of various targeted drugs on ccRCC organoids, such as SU11274, foretinib, cabozantinib, as well as a combination of lenvatinib and everolimus. The ccRCC organoids were sensitive to SU11274 and foretinib, with decreased gene expression of pAKT and pERK. These two genes are important in regulating cell proliferation and stasis. However, only foretinib was able to consistently induce the activation of cleaved caspase-3, suggesting that it can sustainably induce apoptosis in ccRCC organoids. Therefore, organoids serve as a new model for studying targeted therapeutic drugs and their mechanisms of action.

These advancements not only provide new targets and candidate drugs for kidney cancer treatment but also lay the foundation for the realization of precision medicine, showcasing the immense potential of kidney cancer organoid technology in new drug development.

### 4.2 Personalized treatment

Tumor heterogeneity poses a significant challenge in cancer treatment because tumor cells may exhibit different genetic and phenotypic characteristics among different patients, or even within different tumor sites in the same patient. These differences result in significant individual variations in patients’ responses to chemotherapy drugs, emphasizing the importance of personalized treatment plan selection. In this context, kidney cancer organoids, as an innovative technology, offer unique advantages for achieving personalized treatment. By conducting drug sensitivity tests on kidney cancer organoids cultured from specific patient tumor cells, this method can rapidly identify the most effective drugs and combinations for individual patients, optimizing treatment outcomes while reducing ineffective treatments and unnecessary side effects.

To gain a deeper understanding of the drug responsiveness of tumor cells in kidney organ models, researchers have employed techniques such as RNA sequencing and DNA mutation analysis. Specifically, whole exome sequencing has been used to compare gene expression between tumor cells and corresponding normal tissues, while analyzing the allele frequencies of wild-type and mutant genes in tumor cells. The research findings reveal that kidney organ models can reflect the characteristics of primary cancer tissue at a multi-clonal level, despite differences in allele frequencies of about 5% between kidney organ models and actual cancer tissue samples. This indicates that kidney organ models can serve as effective patient models for evaluating tumor cell drug responses, providing an accurate platform for analyzing the sensitivity and resistance of tumor cells to specific drugs.

Through this genomic analysis, doctors can identify key genetic variations in tumor cells, thereby customizing treatment plans and selecting the most effective treatment strategies for patients. This not only facilitates precision medicine but also avoids unnecessary drug resistance in patients due to mismatched medications. Therefore, kidney cancer organoid technology plays a crucial role in advancing personalized cancer treatment, providing physicians with a powerful tool to optimize treatment choices and improve treatment efficacy.

### 4.3 Tumorigenesis and the discovery of tumor markers

In the field of renal cancer research, traditional *in vitro* cell cultures and animal models have provided valuable insights into understanding tumor biology. However, they often fail to comprehensively simulate the multifaceted features of human renal cancer. Recently, the development of renal cancer organoid models has offered a novel platform for exploring tumor initiation mechanisms and identifying potential tumor biomarkers and therapeutic targets. An innovative study combined Renca cells (a mouse renal cell carcinoma cell line) with renal progenitor cells to create such hybrid organoid models, opening up new avenues for gene discovery and functional studies in renal cancer (78).

In this study, the research team initially utilized siRNA-mediated gene silencing technology to target BCL-2/adenovirus E1B 19 kDa protein-interacting protein 3 (Bnip3), gelsolin (Gsn), and caveolin-1 (Cav-1). They observed that this intervention significantly inhibited the growth and migration capabilities of Renca cells. This finding revealed potential key roles of Bnip3, Cav-1, and Gsn in renal cell carcinoma development, providing important clues for subsequent research. Subsequently, by knocking down Bnip3, Cav-1, or Gsn in renal cancer/renal progenitor cell hybrid organoid models, the research team further validated the roles of these genes in renal organogenesis and renal cell carcinoma development. Of note, partially silencing these genes rescued the inhibition of renal developmental processes mediated by Renca cells, indicating their potential regulatory roles in renal cancer initiation.

These results not only demonstrate the renal cancer/renal progenitor cell hybrid organoid model as a powerful tool for studying renal cancer initiation mechanisms and identifying tumor biomarkers but also underscore the value of Bnip3, Cav-1, and Gsn as potential therapeutic targets for renal cancer. Furthermore, the insights provided by this study deepen our understanding of renal cancer biology and offer valuable information for developing treatment strategies targeting specific molecular targets.

In conclusion, the development and application of renal cancer/renal progenitor cell hybrid organoid models not only provide a highly relevant biological platform for studying renal cancer gene function and discovering tumor biomarkers but also guide future research and treatment development in the realm of precision medicine for renal cancer.

### 4.4 Organoid biobank construction

In recent years, with the rapid advancement of precision medicine, organoid technology has emerged as a crucial tool for simulating the biology of human organs and disease mechanisms. Particularly in pediatric oncology research, the creation of renal cancer organoids has provided researchers with an unprecedented platform to delve deeper into the biological characteristics of renal cancer and its responsiveness to treatments. A recent study successfully established the first organoid biobank specifically targeting pediatric renal cancer, representing a groundbreaking development in the field of renal cancer research. This biobank has collected and preserved organoid models of most subtypes of pediatric renal cancer, including Wilms’ tumor, malignant rhabdoid tumors, RCC, and other rare renal tumor entities such as congenital mesoblastic nephroma and post-nephrectomy sarcoma. This comprehensive collection of subtypes not only advances fundamental research into various types of renal cancer but also paves the way for future clinical applications.

These pediatric renal cancer organoids exhibit highly similar characteristics to the primary tumors on multiple levels. Their phenotypic, genetic, epigenetic, and gene expression features align consistently with those of their corresponding natural tumor samples, a fact validated through various molecular biology and histological techniques (37, 79, 80). This high degree of similarity suggests that pediatric renal cancer organoids can serve as effective models for studying tumor biology, screening potential drugs, and devising personalized medical strategies.

Currently, the Human Cancer Model Initiative is developing a globally accessible organoid culture biobank to provide publicly shared data (24). In summary, the establishment of the pediatric renal cancer organoid biobank not only propels the progress of renal cancer research but also provides new strategies and approaches for future clinical treatments. This significant advancement signifies that organoid technology has entered a new phase of application in the field of renal cancer research and treatment, demonstrating vast potential and promising prospects.

## 5 Prospect

### 5.1 Prospects for the construction of organoids in kidney cancer

Tumor organoids, as an *in vitro* tumor model, effectively recapitulate the heterogeneity of tumors, bridging the gap between patient-derived cells (PDCs) and patient-derived xenografts (PDXs). Tumor organoids have been widely utilized in cancer modeling, basic cancer research, and anticancer therapy. The establishment of optimized culture media and co-culture systems reflects a more realistic TME, aiding in our understanding of the intrinsic mechanisms underlying tumor initiation and drug resistance. Moreover, the construction of large-scale organoid banks or biobanks enhances the utility of drug efficacy testing and improves the credibility of drug screening results, offering guidance for future personalized medicine endeavors. However, despite promising prospects, there is still substantial room for improvement.

Currently, research on renal cancer organoids primarily focuses on ccRCC, with limited and less mature efforts directed towards constructing organoids of other types. Therefore, future endeavors to construct organoids of other types will deepen our understanding of renal cancer, benefiting more patients. Another key obstacle is the low success rate of cultivation. Most current methods for constructing renal cancer organoids are based on methods previously described by Grassi et al (52), which are more suitable for cultivating normal kidney organoids and have a significantly lower success rate for cultivating ccRCC organoids. Hence, it is necessary to investigate new methods tailored for renal cancer organoid construction. Additionally, the choice of ECM for organoid construction remains problematic. A significant portion of renal cancer organoids currently utilize Matrigel, sourced from mouse sarcoma tissue, leading to differences in soluble component concentrations between suppliers and batches due to variations in animal sources and serum harvest seasons and geographical locations. Moreover, ECM contains uncertain and xenogenic impurities that may affect organoid phenotype (81). Synthetic hydrogels seem to achieve consistency between batches. Agata et al. successfully constructed ccRCC organoids in collagen matrices, with organoids cultured using this method maintaining structural integrity for up to 21 days without losing their adhesive properties (56).

The lack of multicellular components is still considered a significant limitation of tumor organoids. Developed co-culture techniques for organoids can mimic cancer-stroma cell interactions *in vivo* (82). Furthermore, to mimic mechanical and chemical signals in the *in vivo* microenvironment, microfluidics-based organ chips have become a research hotspot. Organ chips can achieve spatial and temporal manipulation of pH gradients, oxygen concentration gradients, growth factor concentration gradients, nutrient gradients, etc. Complex organ chips can even achieve interconnection of multiple chambers and dynamic fluid circulation to simulate multi-organ communication (83).

However, due to the lack of vascular systems, tumor organoids can only grow to a limited size due to insufficient nutrient supply. Although we can promote tumor angiogenesis by controlling hypoxia and adding vascular endothelial growth factors, the absence of vascular pumps prevents tumor organoids from forming blood flow and circulation. In future research on organ chips, attempts to integrate 3D bioprinting technology to embed bioengineered blood vessels, along with the addition of miniature blood pumping devices externally, will break through these limitations mentioned above.

### 5.2 Prospects for the application of kidney cancer organoids

#### 5.2.1 Gene editing (CRISPR-Cas9)

The application of small molecules and antibodies that specifically target key proteins involved in carcinogenic signaling pathways has significantly improved the survival rate of kidney cancer. However, existing treatment options remain limited and there is a lack of comprehensive understanding of resistance mechanisms. Extensive sequencing programs have revealed that tumor cells in the body often produce resistant cells through stress-induced mutagenesis, leading to tumor recurrence. Consequently, the scientific community has been striving to elucidate the genetic variations of cancer, aiming to enhance the understanding of cancer occurrence and treatment responses. In recent years, the CRISPR/Cas9 system has increasingly been applied in exploring and controlling various diseases, including kidney cancer. The CRISPR/Cas9 gene editing system has demonstrated significant efficacy in identifying cancer genes and assessing drug-gene interactions. By integrating novel ECM, co-culture of cells, and microfluidics technologies, tumor organoids serve as valuable models for studying the cancer microenvironment and facilitating the translation of preclinical findings into clinical efficacy. Takeda and others have utilized the CRISPR-Cas9 functionality combined with human CRC-derived organoids and mouse intestinal tumor organoids to validate the driver genes in CRC (84). Through this platform, they demonstrated that Acvr1b, Acvr2a, and Arid2 can act as tumor suppressors in CRC. They also discovered that the co-occurrence of mutations in activin and transforming growth factor-β receptors synergistically promotes tumorigenesis and elucidated the role of activin receptors. This experimental system can also be applied to kidney cancer organoids, which may further help identify new cancer-driving genes.

Moreover, in 2020, Artegiani and others developed CRISPR-HOT, a novel genetic tool for labeling specific genes in human organoids, initially used to study the division patterns of human hepatocytes (85). CRISPR-HOT is applied to fluorescently label and visualize the subcellular structures of rare intestinal cell types by generating reporter molecules. Some cell types are rare and difficult to study, but CRISPR-HOT can easily label and allow visualization of these cells. Another report from the same group indicated that producing genetically engineered human fetal liver organoids using CRISPR-HOT only takes 2–3 months, which is highly efficient. This new tool can be used for research on cell fate and differentiation, developmental diseases, and can be utilized to visualize any type of gene or cell. CRISPR-HOT also shows promise for advances in cancer research.

#### 5.2.2 Patient-derived organoid xenograft (PDOX) models

In exploring the future directions of kidney cancer treatment, organoid technology and its application in Patient-Derived Organoid Xenograft (PDOX) models have shown tremendous potential. Organoid technology aims to construct *in vitro* models that precisely simulate the tumor microenvironment within an individual, a goal that is challenging yet represents the direction of future advancements in this technology. Currently, despite significant progress in organoid technology, we are still some distance from fully replicating an individual’s *in vivo* tumor model. In this process, the PDOX model offers a viable interim solution. The PDOX model combines the advantages of tumor organoids with PDX models, by transplanting tumor organoids into immunodeficient mice, overcoming the low success rate of PDX model establishment and the issue of scarce tumor samples, while effectively amplifying tumor cells and preserving their phenotype. This method not only helps preserve the histological characteristics, tumor marker expression, and molecular genetics of the primary tumors but also provides a powerful *in vivo* model for drug development and pharmacological research.

Looking ahead, the application of PDOX models in organoid technology will be an important research direction. By further optimizing this model, we hope to make significant advances in disease mechanism research, drug screening, and personalized medicine. For example, by analyzing drug responses in PDOX models, researchers can more accurately predict the effects of drugs in humans, thereby accelerating the discovery and development of effective medications. Additionally, the use of PDOX models can also aid in understanding the mechanisms of tumor resistance to treatments, providing new strategies to overcome this challenge.

In summary, the integration of tumor organoid technology and PDOX models offers new perspectives and methods for cancer research and treatment. By delving deeper into this direction, we can hope to achieve more personalized and precise kidney cancer treatment strategies in the future, bringing greater hope to patients.

## 6 Summary

With technological advancements, organoids are destined to become the mainstream model in biomedical research. Although not yet fully mature, tumor organoids have already played a crucial role in cancer research and therapy. The establishment of optimized culture media and co-culture systems, along with the application of organ chips, reflects a more realistic TME, aiding in our understanding of tumor initiation and drug resistance mechanisms. Additionally, the construction of large-scale organoid banks or biobanks enhances the utilization of drug efficacy testing and improves the credibility of drug screening results, offering guidance for future personalized medicine endeavors (86).
